# Effect of the Preparation Method (Sol-Gel or Hydrothermal) and Conditions on the TiO_2_ Properties and Activity for Propene Oxidation

**DOI:** 10.3390/ma11112227

**Published:** 2018-11-09

**Authors:** Laura Cano-Casanova, Ana Amorós-Pérez, María Ángeles Lillo-Ródenas, María del Carmen Román-Martínez

**Affiliations:** MCMA Group, Department of Inorganic Chemistry and Materials Institute, University of Alicante, E-03080 Alicante, Spain; laura.cano@ua.es (L.C.-C.); ana.amoros@ua.es (A.A.-P.); mcroman@ua.es (M.d.C.R.-M.)

**Keywords:** TiO_2_ synthesis method, HCl, photocatalysis, gas phase, VOCs elimination

## Abstract

Since the two most commonly used methods for TiO_2_ preparation are sol-gel (SG) and hydrothermal (HT) synthesis, this study attempts to compare both methods in order to determine which one is the most suitable to prepare photocatalysts for propene oxidation. In addition, this work studies how the concentration of the HCl used for hydrolysis of the TiO_2_ precursor affects the properties of the obtained materials. Also, the effect of avoiding the post-synthesis heat-treatment in a selection of samples is investigated. The photocatalysts are characterized by XRD, N_2_ adsorption-desorption isotherms and UV-vis spectroscopy, and the study tries to correlate the properties with the photocatalytic performance of the prepared TiO_2_ samples in propene oxidation. TiO_2_ materials with high crystallinity, between 67% and 81%, and surface area (up to 134 m^2^/g) have been obtained both by SG and HT methods. In general, the surface area and pore volume of the TiO_2_-HT samples are larger than those of TiO_2_-SG ones. The TiO_2_-HT catalysts are, in general, more active than TiO_2_-SG materials or P25 in the photo-oxidation of propene. The effect of HCl presence during the TiO_2_ synthesis and of the post synthesis heat treatment are much more marked in the case of the SG materials.

## 1. Introduction

The environmental legislation and related actions show an increasing concern in the elimination of undesired organic air pollutants like volatile organic compounds (VOCs). The removal of VOCs is a topic of great interest since they are very harmful for both the environment and human health, even at low concentration [1,2,3]. VOCs cause alterations in the nervous system and are a source of risk of cancer and genetic mutations [4]. Besides, they are precursors of photochemical oxidants, responsible for acid rain and climate change and take part in the destruction of the ozone layer [5]. Among the different VOCs, propene is object of research since it is present in vehicle emissions, in many industrial effluents, such as petrochemical plants, foundry operations and others, and it is one of the major sources of indoor air pollution, being one of the main components of tobacco smoke [6,7]. As VOCs are usually found at very low concentrations in gas effluents, the implementation of efficient removal techniques is difficult and costly. For this reason, research has been focused on treating gaseous emissions containing VOCs at low concentrations [8,9].

Photocatalysis is an interesting technique for the elimination of VOCs since, on one hand, it generally leads to complete mineralization of the pollutants and, on the other hand, it has the advantage of being applicable to low VOCs concentrations, in contrast to most techniques used to remove these contaminants, such as condensation, absorption or adsorption, that are normally used at higher VOCs concentrations [10,11,12,13,14]. TiO_2_ is one of the most studied photocatalysts because of its high chemical stability and resistance to photo-induced corrosion and because it is an abundant, cheap, not toxic and biocompatible material [15,16]. It can be used alone or forming hybrid catalysts, being one example the synthesis of Cu_2_O/TiO_2_ heterostructures recently reported [17]. However, TiO_2_ presents, as well, some drawbacks (low photoactivity under solar radiation, high rate of e^−^/h^+^ pairs recombination) and many efforts have been made over the last few years to overcome them and improve its efficiency [18,19,20,21].

Many parameters affect the photocatalytic oxidation of pollutants and both the photocatalysts’ properties and the process variables play important roles. The relative importance of these variables is often different depending on the application [16,22], although the vast literature survey performed remarks the importance of: the anatase content [23,24], the presence of small amounts of other crystalline mixed phases [22,25,26] and, especially, the porosity of the photocatalysts [27].

The synthesis method has shown to strongly determine the key physical and chemical properties of TiO_2_ photocatalysts [28,29,30,31]. There are several synthesis methods that differ in the degree of complexity and cost, and whose selection depends on the desired properties of the prepared TiO_2_, the available equipment, the experience of the research group and many others. However, simple, environmentally friendly and low cost methods are obviously preferred. The sol-gel (SG) method has been, up to know, the most widely used because of the low cost of the required equipment, the mild reaction conditions and also because, usually, TiO_2_ of high homogeneity and purity is obtained [32,33,34,35,36,37]. Although the hydrothermal technique is less used, it could also fit well these requirements and, because of that, it is recently receiving a lot of attention as a potential method for preparing highly crystalline TiO_2_ [28,38,39]. In both methods, the nature and concentration of the acid medium used to hydrolyse the titania precursors seems to play an important role in controlling the morphology and crystalline structure of the synthesized TiO_2_ [25,40,41,42,43,44].

In a previous work we faced the preparation of TiO_2_ photocatalysts by one-step hydrothermal synthesis (at low temperature and short time) using HCl of variable concentration as hydrolysis agent and avoiding the use of surfactants [45] (in contrast to many published works that include two steps and the use of surfactants and co-surfactants [40,41,46], or organic solvents, like n-hexane or cyclohexane [41,42,46]). The catalysts were thoroughly characterized, and especial effort was made to determine the TiO_2_ crystalline/amorphous proportion by means of quantifying the amount of the crystalline titania phases present. A detailed study of the textural properties and of the surface chemistry was also carried out. The obtained samples had relatively large surface area (100–135 m^2^/g) and high crystallinity (about 75–81%) and the proportion of anatase, brookite and rutile was influenced by the HCl concentration. The photocatalysts were tested in the low-concentration-propene oxidation and the influence of the catalysts’ properties on the photocatalytic performance was analysed [45]. An interesting conclusion was that most of the prepared TiO_2_ materials were more active for propene oxidation than the well-known P25 titania, being porosity and crystalline composition the two most influencing parameters for this application.

In order to determine if the sol-gel method can render TiO_2_ photocatalysts with the outstanding properties of the hydrothermal samples mentioned above, the present work focuses on the sol-gel preparation of an analogous series of samples. In addition and to complete the previous work, HT and SG samples have also been prepared without acid addition, and the effect of the post-synthesis heat treatment has also been investigated in selected samples. To extract valuable conclusions, all the materials have been characterized in detail and evaluated in the propene oxidation reaction. Thus, the final purpose of our study is to compare the catalytic behaviour of samples obtained in different conditions (preparation method, HCl concentration, post synthesis heat-treatment) for the photooxidation of propene at low concentration, keeping absolutely constant the reaction conditions. For this aim, the two critical features that the study must focus on are proper characterization of materials and evaluation of their photocatalytic activity [47].

Thus, basically, the objective of the present work is to analyse how the synthesis method and the acid medium affect the properties and activity of the photocatalysts. To the best of our knowledge, such a comparison in the same experimental conditions has never been reported before in the literature.

## 2. Materials and Methods

### 2.1. Materials

Titanium (IV) tetraisopropoxide (TTIP, 97%) was purchased from Sigma-Aldrich, St. Louis, MO, USA. Absolute ethanol (C_2_H_6_O, 99.8%) and hydrochloric acid (HCl, 37%) were supplied by Panreac, Barcelona, Spain. All reactants have been used without further purification. The commercial titania P25 from Degussa has been used as reference photocatalyst.

### 2.2. Preparation of TiO_2_ Materials

TiO_2_ was prepared using procedures adapted from the sol-gel method described by Wang et al. [48]. The experimental conditions were chosen with the objective of preparing materials by a one-step synthesis method at mild conditions (low temperature and time) and avoiding the use of surfactants or solvents, such as n-hexane.

Two series of photocatalysts, with five samples each of them, were prepared by sol-gel and hydrothermal synthesis methods, respectively.

In both cases, a mixture prepared with titanium tetraisopropoxide (4 mL) and ethanol (20 mL) was stirred at room temperature, first magnetically (1 h) and then in ultrasonic bath (30 min). Then, a solution containing HCl ((4 mL) 0.5, 0.8, 1 or 12 M) and ethanol (10 mL) was added dropwise, and the mixture left under continuous stirring for 1 h. The synthesis was also performed without HCl, and H_2_O (4 mL) was added instead.

In the case of the sol-gel method, the product of the above described procedure was dried at 100 °C for 12 h. In the case of the hydrothermal synthesis, such a product was transferred to an autoclave and maintained at 180 °C for 12 h and, afterwards, it was dried at 100 °C for 12 h. Finally, both SG and HT samples were heated in air in a muffle at 5 °C/min up to 350 °C and kept for 2 h at this temperature.

The synthesized materials are named TiO_2_-XM-SG and TiO_2_-XM-HT to distinguish the samples prepared by the sol-gel and hydrothermal methods, respectively. In both cases, XM refers to the molar concentration of the hydrochloric acid solution used in the synthesis (0M is used for samples prepared without HCl). Note that the HCl concentration in the synthesis pot was approximately 10 times lower than the one of the added solution (i.e., if 4 mL HCl 12 M was used, considering that the total volume is 38 mL, the HCl concentration in the synthesis medium was 1.27 M).

Several samples (TiO_2_-0M-SG, TiO_2_-0M-HT, TiO_2_-12M-SG and TiO_2_-0.8M-HT) have been prepared twice to study the reproducibility of the preparation method. These samples have also been prepared avoiding the post synthesis heat treatment to analyse the effect of such treatment.

### 2.3. Characterization

The percentage of crystalline TiO_2_, phase composition and crystallite size were determined by X-ray diffraction (XRD) using the analysis method described in a previous work [45]. XRD patterns were recorded both for TiO_2_ samples and for mixtures of these samples with CaF_2_ (50%, w/w) using the equipment Miniflex II Rigaku (30 kV/15 mA) with Cu Kα radiation at a scanning rate of 2°/min, in the 2θ range 6–80°. The average crystallite size, referred to as crystal size, was calculated by the Scherrer equation (Equation (1)):

(1) $$\;B=\frac{K\lambda }{\beta \cos\theta }\;$$

where B is the average crystallite size (nm); λ is the radiation wavelength (0.1540 nm for Cu Kα), K is the Scherrer constant (K = 0.93), β is the full width at half maximum intensity (FWHM) and θ is the angle associated to the main peak of the studied phase (2θ values of 25.3, 27.5 and 30.8° for anatase (A), rutile (R) and brookite (B), respectively).

The porous texture was characterized by N_2_ adsorption-desorption at −196 °C in a volumetric Autosorb-6B apparatus from Quantachrome. The samples were previously degassed at 250 °C for 4 h. The Brunauer, Emmett and Teller (BET) equation was applied to the isotherms to get the apparent BET surface areas (S_BET_) and the Dubinin–Radushkevich equation was used to determine the total micropore volume (V_DR_ N_2_, pores with size < 2 nm) [49]. The mesopore volume (V_meso_ 2 nm< ø < 50 nm) was estimated as the difference between the volume of N_2_ adsorbed at P/P_0_ = 0.9 and P/P_0_ = 0.2, expressed as a liquid [49]. The total pore volume (V_T_) was determined from the volume of nitrogen adsorbed at a relative pressure P/P_0_ = 0.99.

The optical absorption properties were studied by UV-vis/DR spectroscopy (Jasco V-670, Pfungstadt, Germany, with integrating sphere accessory and powder sample holder). BaSO_4_ was used as the reference standard and the reflectance signal was calibrated with a Spectralon standard (Labsphere SRS-99-010, 99% reflectance).

The absorption edge wavelength was estimated from the intercept at zero absorbance of the high slope portion of each individual spectrum in the range 200–800 nm (absorbance method). Then, the band gap can be calculated [50] as:

(2) $$\;\mathrm{Eg}=\frac{1239.8}{\lambda }\;$$

where Eg is the band gap energy (eV) and λ is the edge wavelength (nm).

### 2.4. Photocatalytic Oxidation of Propene

The experimental system used to perform the activity tests was designed in our laboratory and basically consists of a quartz reactor (AFORA, ∅ 1.5 cm) and a 365 nm Philips UV lamp (UV-A) placed parallel to the reactor at a distance of about 1 cm. The assembly reactor-lamp is surrounded by a cylinder covered by tinfoil. A scheme of this system is shown elsewhere [9].

In a typical experiment, 0.11 g of photocatalyst are placed on a quartz wool plug inside the reactor and, after purging with helium, a stream of 100 ppmv propene in air (30 or 60 mL/min (STP)) is passed through the reactor at 25 °C, being the outlet gas continuously analysed by mass spectrometry (Balzers, Thermostar GSD 301 01). After stabilization of the propene signal, m/z = 41, the lamp is switched on and when stationary propene concentration is achieved, the system is kept measuring for a period of about 3 h.

The experiments were repeated at least twice to check reproducibility. Propene conversion is calculated using the expression of Equation (3):

(3) $$\;\mathrm{Propene}\;\mathrm{conversion}\;\left(\%\right)=\frac{C_{\mathrm{initial}\;C3H6}-{\;C}_{\mathrm{stationary}\;C3H6\;}}{C_{\mathrm{initial}\;C3H6}}\times \;100\;$$

where C_initialC3H6_ is the initial propene concentration, 100 ppmv, and C_stationary C3H6_ is the stationary propene concentration reached after a certain time of irradiation.

Carbon dioxide and water produced during oxidation are followed by mass spectrometry, being CO_2_ quantified using a calibrated cylinder (300 ppmv CO_2_ in helium). A mass scan revealed that CO_2_ is the only oxidation product.

## 3. Results and Discussion

### 3.1. Characterization of TiO_2_ Samples

#### 3.1.1. XRD Analysis

The characteristic XRD peaks of the different TiO_2_ phases appear at the following 2θ values [51,52,53]:

- Anatase: 25.3° (101), 37.8° (004), 48.0° (200), 54.5° (105), 55.0° (211), 62.7° (204), 70.4° (116) and 74.5° (220).
- Brookite: 25.3° (120), 25.7° (111) and 30.8° (121).
- Rutile: 27.5° (110), 36.1° (101) and 54.4° (211).

These data indicate that the main diffraction peak of anatase (101) overlaps with the (120) and (111) peaks of brookite.

**Figure 1:**
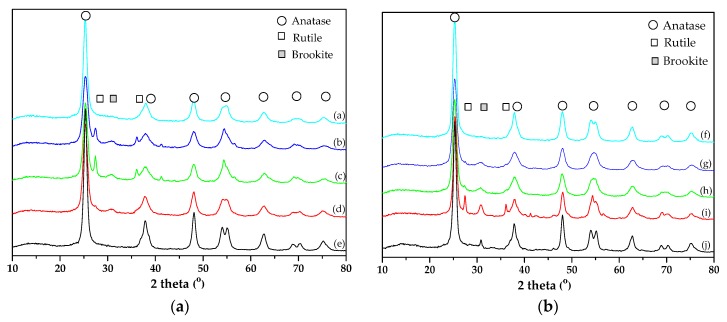
XRD patterns of: (**a**) SG samples ((a) TiO_2_-0M-SG, (b) TiO_2_-0.8M-SG, (c) TiO_2_-1M-SG, (d) TiO_2-_5M- SG and (e) TiO_2-_12M- SG) and (**b**) HT samples ((f) TiO_2_-0M-HT, (g) TiO_2_-0.8M-HT, (h) TiO_2_-1M-HT, (i) TiO_2_-5M-HT and (j) TiO_2_-12M-HT).

Figure 1 shows the XRD patterns obtained for the synthesized TiO_2_ samples, where it can be observed that samples TiO_2_-0.8M, TiO_2_-1M and TiO_2_-5M, both SG and HT, contain anatase, brookite and rutile; sample TiO_2_-12M-SG only contains anatase and sample TiO_2_-12M-HT contains anatase and brookite. The samples prepared without HCl (both SG and HT) contain, as well, only anatase.

**Figure 2:**
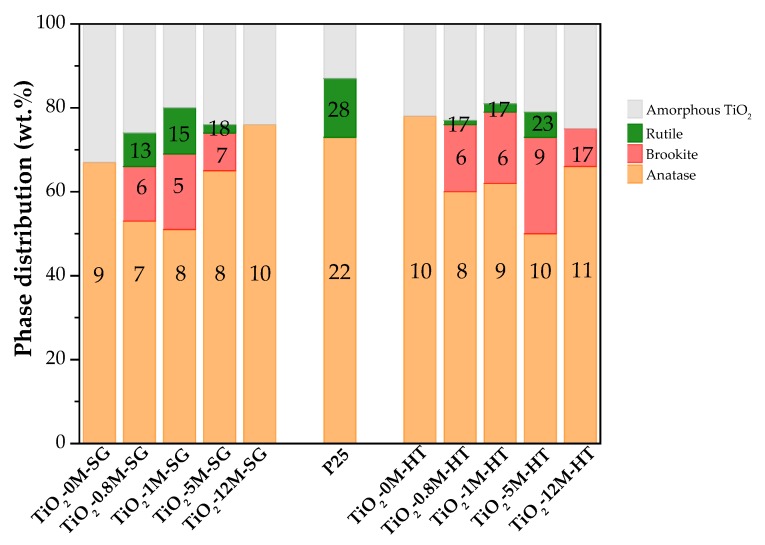
Distribution of crystalline and amorphous phases in TiO_2_-SG and TiO_2_-HT samples. Numbers in the figure refer to the mean crystal size (nm) of each TiO_2_ crystalline phase.

The characterization of crystalline and amorphous TiO_2_ has been object of great interest in the recent literature devoted to photocatalysis [54,55,56,57,58]. In the present work, the method proposed by Cano-Casanova et al. [45] has been selected for crystalline/amorphous characterization considering the multiphasic nature and the presence of brookite in most photocatalysts. The parameters calculated from the XRD data of Figure 1, amount (in wt.%) of the different TiO_2_ crystalline phases, amorphous TiO_2_ fraction (in wt.%) and average crystal size of each crystalline phase are graphically presented in Figure 2 (and also in Table S1, see Supplementary Information).

The proportion of amorphous phase determined for P25 titania (13%) is similar to that found in the literature [56,59,60,61], what supports the reliability of the analysis carried out.

Figure 2 shows that the content of amorphous TiO_2_ in most of the prepared samples ranges between 19% and 26% (see Table S1), which indicates that, in general, the preparation method and HCl concentration do not significantly affect the degree of crystallinity reached. Only in sample TiO_2_-0M-SG the content of amorphous TiO_2_ is slightly higher, 33%, and it must be mentioned that this sample is grey coloured, in contrast to the rest of samples that are white (see Figure S1 in the Supplementary Information). This result seems to indicate that, although both methods render TiO_2_ with a crystallinity degree above 65%, the presence of HCl is necessary in the sol-gel one to enhance crystallization. However, under hydrothermal conditions (180 °C, 12 h) the crystallization process is more controlled and the absence of HCl is less significant.

As mentioned above, TiO_2_-0M-SG sample only contains anatase (Figure 2) and when HCl 0.8M, 1M or 5M are used in the synthesis, the amount of anatase decreases and brookite and rutile phases appear. The proportion of the three phases varies with the concentration of the HCl used, being TiO_2_-1M-SG the sample with the largest amount of brookite and rutile. It should be pointed out that the only crystalline phase in sample TiO_2_-12M-SG is anatase, as in TiO_2_-0M-SG, and the anatase content is the highest.

In the HT series, also the TiO_2_-0M-HT sample contains only anatase, whereas when 0.8M, 1M or 5M HCl are used in the synthesis, the amount of anatase decreases and brookite and rutile phases appear (Figure 2). However, in contrast with the SG series, catalyst TiO_2_-12M-HT contains a mixture of anatase and brookite. In this case, samples TiO_2_-5M-HT and TiO_2_-0M-HT are those with the lowest and the highest anatase content, respectively.

Figure 2 shows that TiO_2_-5M-HT and TiO_2_-1M-SG samples have some similarities, being these two photocatalysts those containing the highest proportion of brookite and rutile. In addition, data show that both, the presence of HCl and its concentration in the synthesis pot, influence the development of the titania crystalline phases, being this effect different for the two preparation methods. The HT method allows to obtain higher anatase content using low-concentrated HCl, while a pure anatase sample is obtained by sol-gel method using 12 M HCl. It should also be highlighted that the anatase content in samples TiO_2_-12M-SG and TiO_2_-0M-HT is higher than in P25 titania (Figure 2).

In general, the hydrothermal method leads to more crystalline samples, with a slightly larger average crystal size, than the sol-gel one.

The average crystal size (Figure 2 and Table S1) of all the crystalline phases in the two series of samples slightly increases with increasing the HCl concentration used in the synthesis. The anatase crystal size is larger in samples synthesized without HCl, with the exception of sample TiO_2_-12M-SG.

#### 3.1.2. Textural Properties

**Figure 3:**
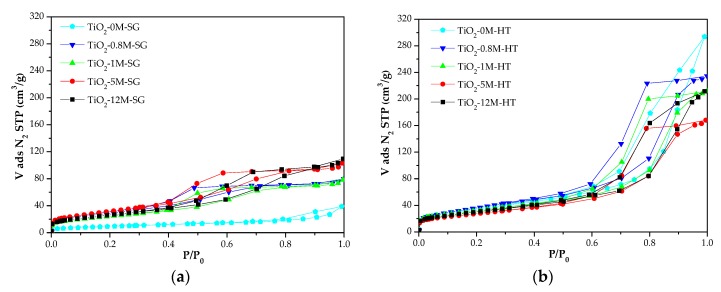
N_2_ adsorption-desorption isotherms at −196 °C of samples: (**a**) TiO_2_-XM-SG and (**b**) TiO_2_-XM-HT.

Figure 3 shows the N_2_ adsorption-desorption isotherms of all synthesized materials. It can be observed that they present type IV isotherms according to the International Union of Pure and Applied Chemistry (IUPAC) classification [62], associated with mesoporous solids, and that the HCl concentration influences the textural properties.

Table 1 presents the textural properties of TiO_2_-XM-SG and TiO_2_-XM-HT samples and of P25 titania. The procedure used to calculate the parameters has been described in the characterization section and reproducibility has been tested in several samples. Results in Table 1 show that the surface areas of the SG samples are slightly lower than those of HT samples (34 to 120 m^2^/g vs. 100 to 134 m^2^/g) and all prepared materials have larger surface area than P25, with the exception of sample TiO_2_-0M-SG. It has been confirmed that the low surface area of sample TiO_2_-0M-SG is a reproducible result.

Samples of both series, excepting TiO_2_-0M-SG, have similar micropore volumes (V_DR_ N_2_), between 0.03 and 0.05 cm^3^/g, but a significant difference is observed in the mesopore volumes. The V_meso_ values of TiO_2_-XM-HT materials are more than twice those of the TiO_2_-XM-SG materials (0.20–0.29 cm^3^/g vs. 0.07–0.10 cm^3^/g). As a consequence, this important difference is also observed in the total pore volume (Table 1).

In the SG series there is no clear relationship between the specific surface area and the concentration of the acid used in TiO_2_ synthesis while, in general, the surface area of the HT photocatalysts decreases when the HCl concentration increases (only sample TiO_2_-12M-HT does not follow the trend).

**Figure 4:**
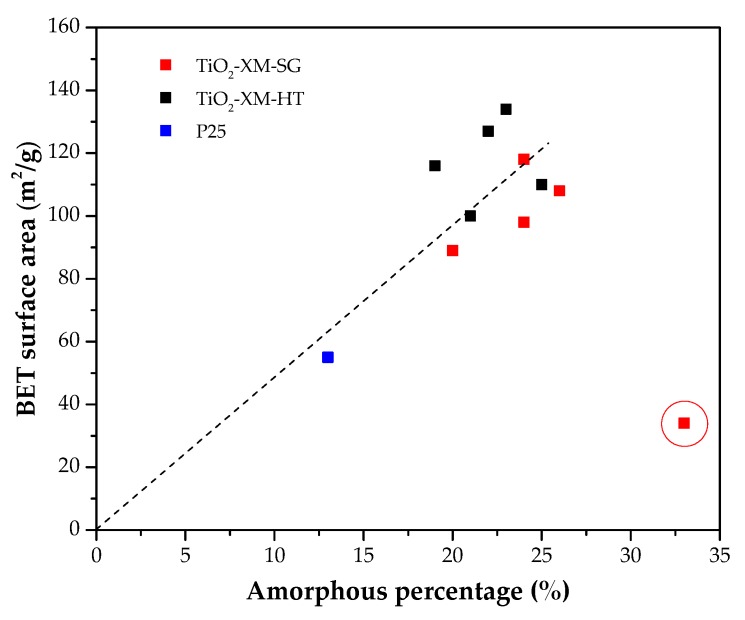
BET surface area vs amorphous percentage for the SG and HT photocatalysts and P25 titania.

A plot of the BET surface area versus the amount of amorphous titania (Figure 4) shows an acceptable positive slope linear trend, in which sample TiO_2_-0M-SG constitutes a clear exception (red circle). Data in Figure 4 also show that, for a similar amount of amorphous phase, the HT samples have higher surface area. Thus, although both methods render TiO_2_ with acceptable crystallinity, these results seem to indicate that in absence of acid, hydrolysis-condensation reactions occur easily during the HT synthesis, while in the SG method the presence of acid is necessary to obtain samples with high surface area because of the role of HCl as catalyst for the alkoxide hydrolysis in these conditions [40,41,42,63].

Attention should also be paid to the relationship between surface area and phase composition. As reported in the literature, higher brookite and rutile contents result in smaller surface areas [64,65]. This could explain the differences in the specific surface area of samples TiO_2_-0.8M-SG and TiO_2_-1M-SG (108 and 89 m^2^/g, respectively, with 18% B and 11% R versus 13% B and 8% R, (Table S1 and Figure 2)). Analogously, the larger specific surface area of TiO_2_-5M-SG (118 m^2^/g) would be related to its lower brookite and rutile content (9% B and 2% R). Although sample TiO_2_-12M-SG does not contain neither brookite nor rutile, its surface area is lower than that of sample TiO_2_-5M-SG, what can be explained considering that the former has larger anatase crystal size, which is consistent with a smaller surface area [66]. 

In the HT series, the low specific surface area of sample TiO_2_-5M-HT can be attributed to its high brookite and rutile content. The difference in the surface area of TiO_2_-0.8M-HT and TiO_2_-1M-HT samples (134 versus 116 m^2^/g), which have similar phase composition, can be attributed to the higher crystallinity of the second one. The surface area of TiO_2_-12M-HT is lower than expected considering its phase composition, which must be a consequence of its relatively large mean crystal size. This can be also the case of TiO_2_-0M-HT sample, that contains anatase (78%), but presents slightly lower surface area than that of TiO_2_-0.8M-HT (60% A, 16% B, 1% R). It should be again highlighted that, in absence of acid, the experimental conditions of the HT method are sufficient for TiO_2_ to be properly synthesized-crystallized.

Comparing the textural properties of samples prepared by SG and HT methods, it can be concluded that, in general, the second one allows obtaining TiO_2_ with larger surface aresa and pore volumes.

#### 3.1.3. Determination of the Band Gap Energy

Table 2 contains the values of the energy band gap (Eg) determined for all the photocatalysts as explained in the characterization section. The energy band gap values of the three titania crystalline phases reported in the literature (determined also by the absorbance method (see the experimental section)) are 3.20, 3.15 and 3.00 eV for anatase, brookite and rutile, respectively [65,66].

In general, data in Table 2 show that Eg values of samples that only contain anatase (TiO_2_-0M-SG, TiO_2_-12M SG and TiO_2_-0M-HT) are lower than the one reported for pure anatase [65]. This could be explained considering that these materials contain a certain percentage of amorphous phase, in the range of 20–33% [67,68]. This can also affect the Eg values of the other samples. For instance, the Eg values of TiO_2_-0.8M-SG, TiO_2_-1M-SG and TiO_2_-5M-SG samples, containing anatase, brookite and rutile (see Figure 2 and Table S1), are relatively close to each other (2.92, 2.80 and 2.95 eV, respectively), but also clearly lower than those indicated above for the pure phases (even considering the proportion of each of them in the sample). Apart from the potential effect of the amorphous titania, the interaction between phases could lead to the modification of the photocatalysts’ band structures [40]. For example, the lowest Eg value corresponds to TiO_2_-1M-SG, which has the largest brookite and rutile contribution.

The same happens in the case of analogous samples of the HT series. Samples TiO_2_-0.8-HT and TiO_2_-1M-HT, which have the same Eg value, have a very similar phase distribution (Figure 2 and Table S1) and, again, the lower Eg value corresponds to the sample with the highest content of brookite and rutile (TiO_2_-5M-HT). Finally, TiO_2_-12M-HT sample, that contains anatase and a small proportion of brookite, shows the highest Eg value, 3.15 eV, almost the same as the sample composed of pure anatase, indicating that the effect of brookite is less important than that of rutile.

**Figure 5:**
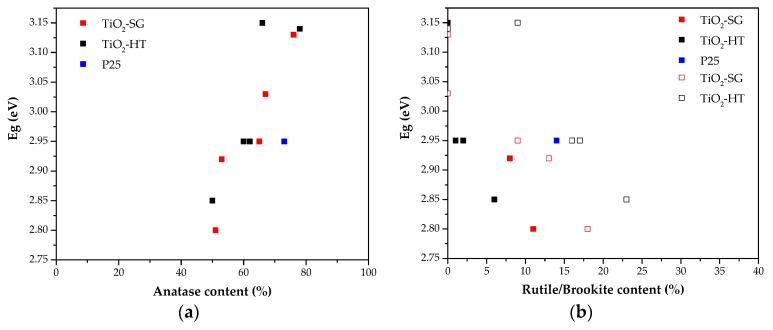
Band gap energy values for SG and HT photocatalysts: (**a**) versus anatase content and (**b**) versus the content of rutile (filled symbols) or brookite (empty symbols).

Figure 5 shows the plot of Eg (eV) versus the anatase content (%), Figure 5a, or versus the rutile or brookite content, Figure 5b, for TiO_2_-XM-SG and TiO_2_-XM-HT samples. In general, the obtained data show that the Eg values seem to be directly related with the anatase contribution. Therefore, samples with similar anatase contents show similar energy band gap values. In the case of rutile/brookite phase contents, the increase in the contribution of either rutile or brookite leads to Eg decrease, although the trend seems to be less linear than in the case of anatase.

These data show that the optical properties of the synthesized materials are very similar to those obtained for the reference P25 titania. The variation found in the Eg values between samples within the respective series of samples (SG and HT) seems to be determined by the phase distribution, the anatase content and the presence of amorphous TiO_2_ [40]. Finally, comparing samples prepared with the same HCl concentration by the two methods, it can be concluded that there are no significant differences in their optical properties.

### 3.2. Photocatalytic Activity

**Figure 6:**
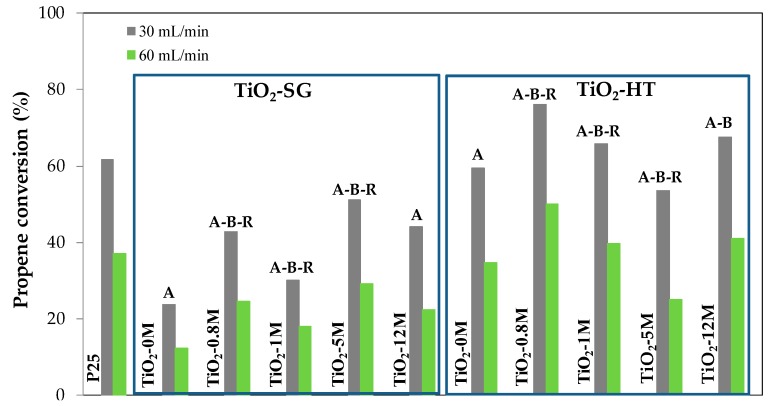
Propene conversion (%) at 30 and 60 mL/min for the SG and HT photocatalysts and for P25.

Figure 6 shows propene conversion data obtained with the SG and HT catalysts and with P25 in experiments carried out with two gas flow rates (30 and 60 mL/min). In general, the same trend in the conversion values is observed for both flow rates, which can be considered as an indication of the reproducible behaviour of these photocatalysts. As expected, propene conversion is higher when the gas flow is lower (the contact time is larger and/or the amount of propene molecules to be oxidized per unit of time is lower).

The carbon balance (propene consumed and CO_2_ formed) confirms that total mineralization of oxidized propene takes place, according to the following reaction and to the literature [9,69]:

2C_3_H_6_ + 9O_2_ → 6CO_2_ + 6H_2_O

Data in Figure 6 show that the SG photocatalysts are less active than P25, while most of the HT samples are more active than the commercial titania and, thus, more active than the analogous SG photocatalysts.

**Figure 7:**
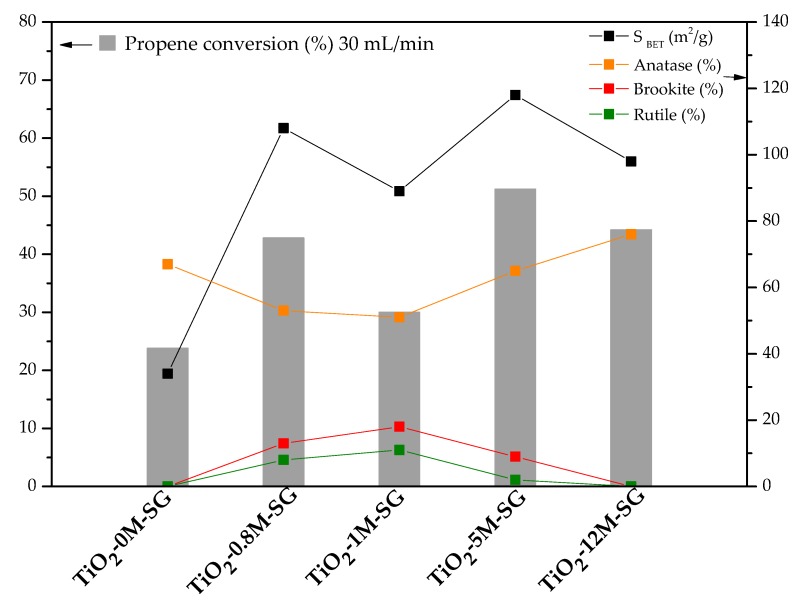
Propene conversion (using 30 mL/min gas flow), surface area and weight percentage of each crystalline phase in the SG photocatalysts.

The activity order in the SG series is the following: TiO_2_-5M-SG > TiO_2_-12M-SG > TiO_2_-0.8M-SG > TiO_2_-1M-SG > TiO_2_-0M-SG. Conversion values and the main properties of SG samples have been plotted in Figure 7.

In general, Figure 7 shows that the activity trend correlates very well with the values of the BET surface, and less markedly with the amount of anatase. For example, the high activity of sample TiO_2_-5M-SG may be explained considering that it has the largest surface area, a high anatase content and small brookite and rutile contents. Thus, the increase in surface area influences positively the activity, and the higher the anatase content and the lower the brookite and rutile contents, the higher the photocatalytic activity obtained. TiO_2_-0M-SG sample shows the lowest propene conversion, which is consistent with its much lower surface area, although it has a relatively high anatase content.

**Figure 8:**
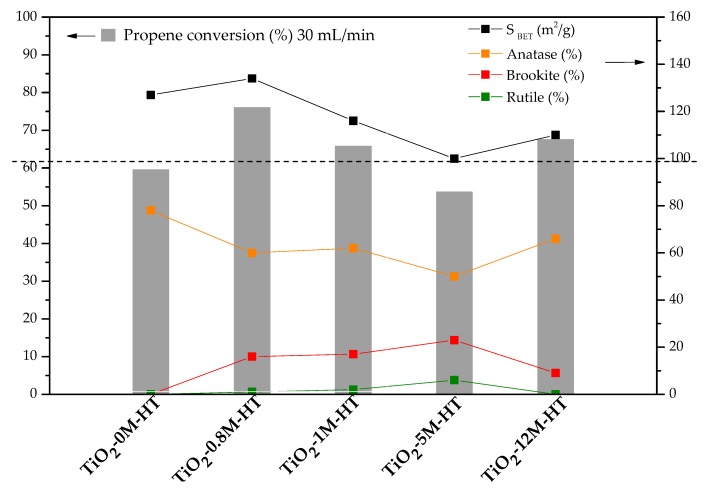
Propene conversions (30 mL/min), surface areas and weight compositions of crystalline phases for the HT-materials.

In the HT series the order of activity is the following: TiO_2_-0.8M-HT > TiO_2_-12M-HT > TiO_2_-1M-HT > TiO_2_-0M-HT > TiO_2_-5M-HT, being TiO_2_-0.8M-HT, TiO_2_-12M-HT and TiO_2_-1M-HT more active than P25. As explained in our previous work [45], and as it happens in the case of SG samples, the trend in catalytic activity is broadly the same than that of surface area and anatase content (Figure 8). It should also be noted that sample TiO_2_-0M-HT is less active than TiO_2_-1M-HT or TiO_2_-12M-HT, in spite of the larger surface area and anatase content of the first one. This result could indicate that the presence of a small content of mixed-phases (i.e., B or B-R) would help to decrease the electron-hole recombination rate, in agreement with previously published results [70,71,72].

One of the main general conclusions that can be extracted is that some parameters, such as surface area and anatase content positively influence the activity, in agreement with the literature [23,24,27]. In contrast, in the conditions studied, differences in the electronic structure of titania seem to play a minor role, also in agreement with previously published results [45,73,74]. 

Paying more attention to phase composition, the most interesting option seems to be based on predominant anatase-based samples, with low brookite and rutile contents, although it is indeed difficult to get to a conclusion regarding this point due the large number of interconnected parameters influencing the photocatalytic activity.

As mentioned, TiO_2_-HT photocatalysts lead to higher propene conversion than TiO_2_-SG ones, which can be justified considering that they have, in general, larger surface areas (100–134 m^2^/g in HT samples versus 90–120 m^2^/g in SG ones). The comparison of SG and HT photocatalysts prepared using HCl of the same concentration (i.e., TiO_2_-0.8 SG and TiO_2_-0.8 HT) shows that, in general, the higher activity of the TiO_2_-HT samples correlates well with their larger specific surface area. The pair TiO_2_-5M SG and TiO_2_-5M HT constitutes an exception, as the activity of both samples is very similar. Other parameters not analysed in this study, such as surface chemistry, might also play a role.

### 3.3. Effect of Avoiding Post-Synthesis Heat Treatment

The results from previous sections correspond to samples submitted to a post-synthesis heat treatment at 350 °C, carried out with the purpose of improving the crystallinity of TiO_2_. However, as hydrothermal synthesis seems to enhance the final crystallinity, it would be interesting to study which are the properties and catalytic behaviour of samples prepared by the SG and HT methods avoiding such heat treatment.

Thus, some additional photocatalysts have been prepared (with and without HCl) avoiding the post synthesis heat treatment (TiO_2_-0M-SG, TiO_2_-0M-HT, TiO_2_-0.8M-HT and TiO_2_-12M-SG). In addition to the two samples prepared without HCl, TiO_2_-0.8M-HT has been chosen because it is the most active sample when treated at 350 °C and TiO_2_-12M-SG was selected because, among the photocatalysts prepared with HCl, it contains the highest amount of anatase from the previous set of samples. The nomenclature of non-treated samples includes WT (“without heat treatment”). Table 3 compiles the properties and propene conversion of TiO_2_-0M-SG-WT, TiO_2_-0M-HT-WT, TiO_2_-0.8M-HT-WT and TiO_2_-12M-SG-WT and of the analogous samples treated at 350 °C, whose names include for better comparison the temperature of the heat treatment, “350”.

TiO_2_-0M-SG-WT sample shows a very high surface area (450 m^2^/g), but it is completely amorphous (XRD spectra is shown in Figure S2, in Supplementary Information), which justifies the low propene conversion obtained (6%). After the heat treatment its crystallinity is developed, but the surface area is strongly reduced and, thus, sample TiO_2_-0M-SG-350 shows a moderate activity.

Surface area (131 m^2^/g) and crystallinity (75%) for TiO_2_-0M-HT-WT sample are high and very similar to that for TiO_2_-0M-HT-350, meaning, in principle, that the heat treatment in this case would almost have no effect. The similarity between propene conversions for both samples, TiO_2_-0M-HT-WT and TiO_2_-0M-HT-350, confirms the relationship between properties and activity.

As previously commented, the SG procedure in absence of HCl does not allow hydrolysis-condensation reactions to take place properly and, thus, the formation of crystalline TiO_2_ becomes hindered. Hence, in this case, such post-heat treatment is necessary to develop crystallinity in SG sample. In contrast, the heat treatment in HT samples does not improve the high crystallinity attained during the synthesis.

This is also observed in the case of TiO_2_-0.8M-HT-WT and TiO_2_-0.8M-HT-350, which have very similar properties and photocatalytic behaviour. Thus, even without heat treatment, the sample prepared with a low HCl concentration presents high crystallinity (70%), containing anatase, brookite and rutile, large surface area (147 m^2^/g) and low crystal size (Anatase-7 nm).

Finally, the crystallinity of TiO_2_-12M-SG-WT is 51% (pure anatase), lower than that for sample TiO_2_-12M-SG-350 (76%), which remarks that by the SG method, even using a high HCl concentration, the development of crystallinity is hindered.

These results indicate that both the heat treatment and the HCl concentration have an important effect on the properties of photocatalysts prepared by the SG synthesis method. Therefore, it can be concluded that adding HCl during the synthesis, both in SG and HT methods, improves the properties and the photocatalytic performance (Figure 7 and Figure 8), although this influence is much more marked for SG materials. Regarding heat treatment, in the SG procedure the post-heat treatment is necessary to develop crystallinity, whereas the heat treatment in HT samples affects only slightly. It must be recalled that higher crystallinity is not always linked to higher propene conversion, since it can be linked to an increase in the mean crystal size and, therefore, to a decrease in the surface area. Anyway, active TiO_2_ photocatalysts (with higher propene conversion than commercial P25) can be obtained by the HT method, without using acid and without a post-synthesis heat treatment.

## 4. Conclusions

TiO_2_ photocatalysts have been prepared by sol-gel (SG) and hydrothermal (HT) methods under mild conditions. The effect of synthesis method, the presence/absence and the concentration of HCl as hydrolysis medium and the post-synthesis heat-treatment on the properties and activity of the photocatalysts have been analysed.

When HCl is used, its concentration influences the development of TO_2_ crystalline phases, being the effect different in SG and HT methods. The HT method leads, in general, to higher crystallinity and allows to obtain a larger amount of anatase using a low HCl concentration. In fact, TiO_2_-0M-HT contains the highest amount of anatase. In the SG method the presence of acid is necessary to enhance the crystallization and to obtain samples with acceptable surface area.

Both SG and HT methods are suitable to obtain photocatalysts with large surface area (larger than that of P25titania) and, in general, TiO_2_-HT samples have larger surface areas and pore volumes than TiO_2_-SG ones.

In both SG and HT series, the Eg values seem to be determined by the phase composition, in particular by the anatase content, the presence of amorphous TiO_2_ and the rutile and brookite contents (being the effect of brookite less important than that of rutile). However, the observed differences in the band gap energies do not seem to affect the photocatalytic performance in this application.

In general, TiO_2_-HT catalysts are more active than TiO_2_-SG ones and P25 titania for propene photo-oxidation. Surface area and anatase content seem to be the parameters that most significantly influence the photocatalytic activity for the studied application. Samples with a high surface area and a high anatase content, probably with a small percentage of brookite-rutile, seem to be the most interesting photocatalysts. The combination of phases could lead to a decrease in electron-hole recombination rate compared to the case of pure anatase samples.

The post synthesis heat treatment of the SG samples is necessary to develop crystallinity, particularly when no HCl is used in the synthesis, whereas the HT method leads to a high crystallinity even without the use of HCl in the synthesis and without the post synthesis heat treatment.

## Figures and Tables

**Table 1:** Textural properties of the SG and HT series of samples.

Sample	S_BET_ (m^2^/g)	V_DR_ N_2_ (cm^3^/g)	V_meso_ (cm^3^/g)	V_T_ (cm^3^/g)
TiO_2_-0M-SG	34	0.01	0.04	0.06
TiO_2_-0.8M-SG	108	0.04	0.07	0.12
TiO_2_-1M-SG	89	0.03	0.08	0.11
TiO_2_-5M-SG	118	0.04	0.10	0.16
TiO_2_-12M-SG	98	0.04	0.10	0.17
TiO_2_-0M-HT	127	0.05	0.38	0.45
TiO_2_-0.8M-HT	134	0.05	0.29	0.36
TiO_2_-1M-HT	116	0.04	0.25	0.33
TiO_2_-5M-HT	100	0.04	0.20	0.26
TiO_2_-12M-HT	110	0.04	0.26	0.32
P25	55	0.02	0.07	0.18

**Table 2:** Energy band gap for SG and HT photocatalysts and for P25 titania.

Sample	Eg (eV)
TiO_2_-0M-SG	3.03
TiO_2_-0.8M SG	2.92
TiO_2_-1M SG	2.80
TiO_2_-5M SG	2.95
TiO_2_-12M SG	3.13
TiO_2_-0M-HT	3.14
TiO_2_-0.8M HT	2.95
TiO_2_-1M HT	2.95
TiO_2_-5M HT	2.85
TiO_2_-12M HT	3.15
P25	2.95

**Table 3:** Physico-chemical properties and propene conversions for TiO_2_-0M-SG, TiO_2_-0M-HT, TiO_2_-0.8M-HT and TiO_2_-12M-SG (with and without heat treatment) and for P25.

Sample	S_BET_ (m^2^/g)	Crystalline TiO_2_ (wt.%)	Average Anatase Crystallite Size (nm)	Propene Conversion (%)
TiO_2_-0M-SG-WT	450	-	-	6.0
TiO_2_-0M-SG-350	34	A (67)	9.0	24.0
TiO_2_-0M-HT-WT	131	A (75)	9.0	70.0
TiO_2_-0M-HT-350	127	A (78)	10	60.0
TiO_2_-0.8M-HT-WT	147	A (53)-B (16)-R (1)	7.0	78.0
TiO_2_-0.8M-HT-350	134	A (60)-B (16)-R (1)	8.0	76.0
TiO_2_-12M-SG-WT	160	A (51)	5.0	68.0
TiO_2_-12M-SG-350	98	A (76)	10.0	44.0
P25	55	A (73)	22	61.5

## Supplementary Materials

The following information is available online at http://www.mdpi.com/1996-1944/11/11/2227/s1, Figure S1: Image of the samples TiO_2_-0M-HT (left) and TiO_2_-0M-SG (right), both treated at 350 °C, Figure S2: XRD patterns of: (a) TiO_2_-0M-SG-WT sample and (b) comparison of TiO_2_-0M-SG-WT with TiO_2_-0M-SG-350, Table S1: Amount (in wt.%) of the different TiO_2_ crystalline phases and of amorphous TiO_2_ and average crystal size for each crystalline phase.
