# Supplementary material for: Effect of the Preparation Method (Sol-Gel or Hydrothermal) and Conditions on the TiO2 Properties and Activity for Propene Oxidation
Source: Materials (Basel). 2018 Nov 9;11(11):2227. doi: 10.3390/ma11112227 (PMC6266794; doi:10.3390/ma11112227)
Supplement: Supplementary file 1 [file materials-11-02227-s001.pdf]

materials

MDPI

*Article*

# Effect of the Preparation Method (Sol-gel or Hydrothermal) and Conditions on the $TiO_2$ Properties and Activity for Propene Oxidation

**Laura Cano-Casanova, Ana Amorós-Pérez, María Ángeles Lillo-Ródenas * and María del Carmen Román-Martínez**

MCMA Group, Department of Inorganic Chemistry and Materials Institute, University of Alicante, E-03080 Alicante, Spain; laura.cano@ua.es (L.C.-C.); ana.amoros@ua.es (A.A.-P.); mcroman@ua.es (M.C.R.-M.)

* Correspondence: mlillo@ua.es; Tel.: +34965903545; Fax: +34965903454

**Table S1.** Amount (in wt.%) of the different $TiO_2$ crystalline phases and of amorphous $TiO_2$, and average crystal size for each crystalline phase.

| Sample | Crystalline Contribution | | | Amorphous Contribution (%) | Average Crystallite Size (nm) | | |
|---|---|---|---|---|---|---|---|
| | A (%) | B (%) | R (%) | | A | B | R |
| $TiO_2$-0M-SG | 67 | - | - | 33 | 9 | - | - |
| $TiO_2$-0.8M-SG | 53 | 13 | 8 | 26 | 7 | 6 | 13 |
| $TiO_2$-1M-SG | 51 | 18 | 11 | 20 | 8 | 5 | 15 |
| $TiO_2$-5M-SG | 65 | 9 | 2 | 24 | 8 | 7 | 18 |
| $TiO_2$-12M-SG | 76 | - | - | 24 | 10 | - | - |
| $TiO_2$-0M-HT | 78 | - | - | 22 | 10 | - | - |
| $TiO_2$-0.8M-HT | 60 | 16 | 1 | 23 | 8 | 6 | 17 |
| $TiO_2$-1M-HT | 62 | 17 | 2 | 19 | 9 | 6 | 17 |
| $TiO_2$-5M-HT | 50 | 23 | 6 | 21 | 10 | 9 | 23 |
| $TiO_2$-12M-HT | 66 | 9 | - | 25 | 11 | 17 | - |
| P25 | 73 | - | 14 | 13 | 22 | - | 28 |

A = Anatase, B = Brookite and R = Rutile.

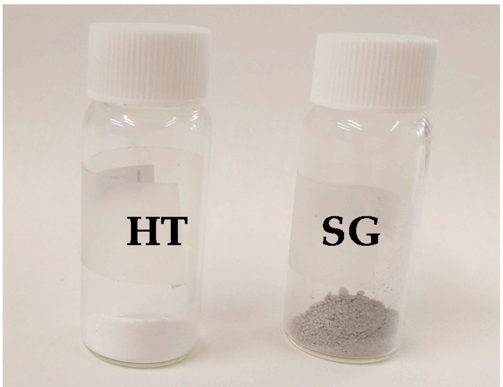


**Figure S1.** Image of the samples $TiO_2$-0M-HT (left) and $TiO_2$-0M-SG (right), both treated at 350 °C.

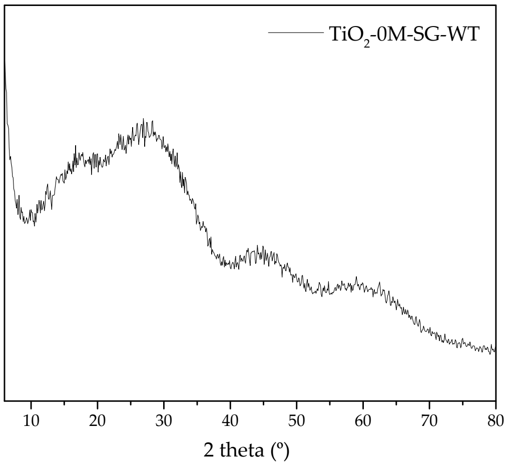


(**a**)

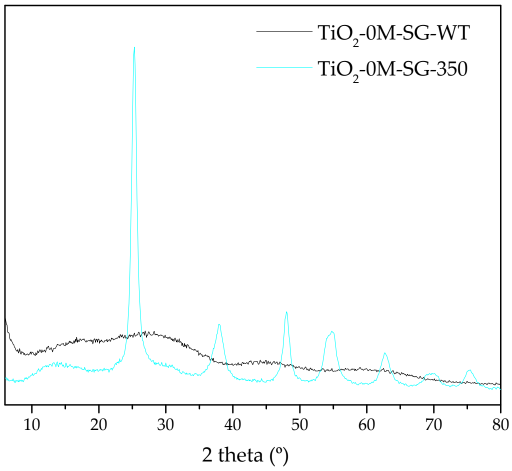


(**b**)

**Figure S2.** XRD patterns of: (**a**) $TiO_2$-0M-SG-WT sample and (**b**) comparison of $TiO_2$-0M-SG-WT with $TiO_2$-0M-SG-350.

© 2018 by the authors. Submitted for possible open access publication under the terms and conditions of the Creative Commons Attribution (CC BY) license (http://creativecommons.org/licenses/by/4.0/).
